# Protein-losing gastroenteropathy complicated with asymptomatic primary biliary cholangitis, refractory to immunosuppressant, and improved by *Helicobacter pylori* eradication: a case report

**DOI:** 10.1186/s12876-022-02170-8

**Published:** 2022-03-07

**Authors:** Misako Tanaka, Hideto Kawaratani, Ryuichi Noguchi, Aritoshi Koizumi, Akihiko Shibamoto, Kosuke Kaji, Naotaka Shimozato, Kuniyuki Kojima, Yoshiaki Nishimura, Hitoshi Yoshiji

**Affiliations:** 1 Internal Medicine, Heisei Memorial Hospital, Kashihara, Nara Japan; 2 Department of Gastroenterology, Nara Medical University, Kashihara, Nara 634-8522 Japan

**Keywords:** Protein-losing gastroenteropathy, *H. pylori* infection, Eradication, Case report

## Abstract

**Background:**

Protein-losing gastroenteropathy (PLGE) is a syndrome with a chief complaint of hypoalbuminemia, which occurs due to plasma protein leakage in the gastrointestinal tract, leading to general edema, ascites, and pleural effusions.

**Case presentation:**

A 71-year-old woman visited another hospital for evaluation of hypoalbuminemia and systemic edema. She was hospitalized for a close inspection of hypoalbuminemia and was diagnosed with PLGE. Steroid and azathioprine therapy was prescribed; however, hypoalbuminemia did not improve, and the patient’s condition worsened due to anasarca. As hospitalization was prolonged, the patient was transferred to our hospital. She was infected with *Helicobacter pylori*, and we performed *H. pylori* eradication. Following *H. pylori* eradication, her edema improved remarkably.

**Conclusion:**

We present the first case wherein *H. pylori* eradication successfully improved protein leakage in the lower gastrointestinal tract in a patient diagnosed with PLGE complicated with refractory to immunosuppressant treatment. *H. pylori* eradication should be considered in patients with PLGE complicated with *H. pylori* infection, without specific endoscopic finding or refractory to immunosuppressants.

## Background

Protein-losing gastroenteropathy (PLGE) is a syndrome with a chief complaint of hypoalbuminemia, which occurs due to plasma protein leakage in the gastrointestinal tract, leading to general edema, ascites, and pleural and pericardial effusions [1]. Various disorders are known to be associated with PLGE. Among these, systemic lupus erythematosus (SLE) and rheumatoid arthritis (RA) have been recognized as relatively common causes of PLGE in autoimmune diseases [2]. Menetrier's disease is known as a PLGE associated with *Helicobacter pylori* (*H. pylori*) infection, and it is reported that *H. pylori* eradication can successfully treat Menetrier’s disease; however, the relationship between PLGE and *H. pylori* infection is yet to be elucidated. In this study, we present the first case of asymptomatic primary biliary cholangitis (aPBC) that responded to *H. pylori* eradication therapy and improved protein leakage in the lower gastrointestinal tract.

## Case presentation

**Fig. 1:**
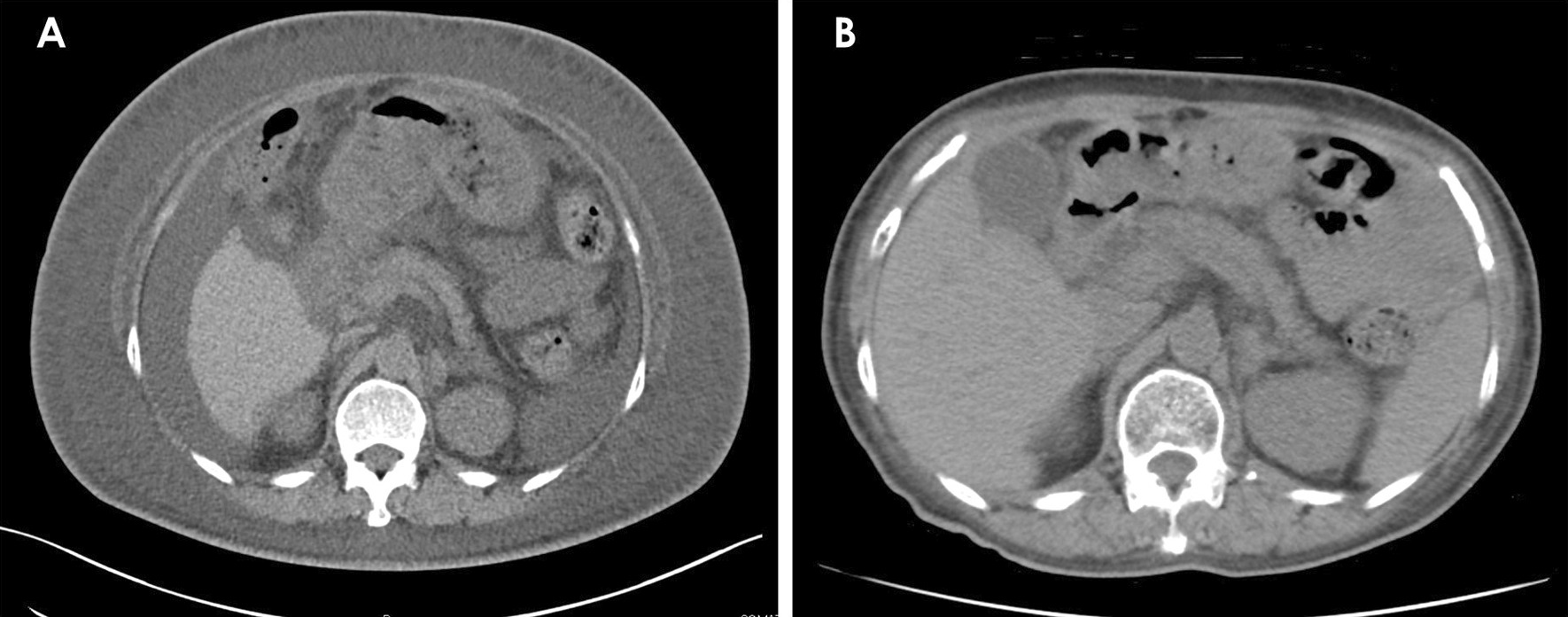
**A** Computed tomography showed subcutaneous edema and ascites. **B** After *Helicobacter pylori* eradication, subcutaneous edema and ascites were improved

**Fig. 2:**
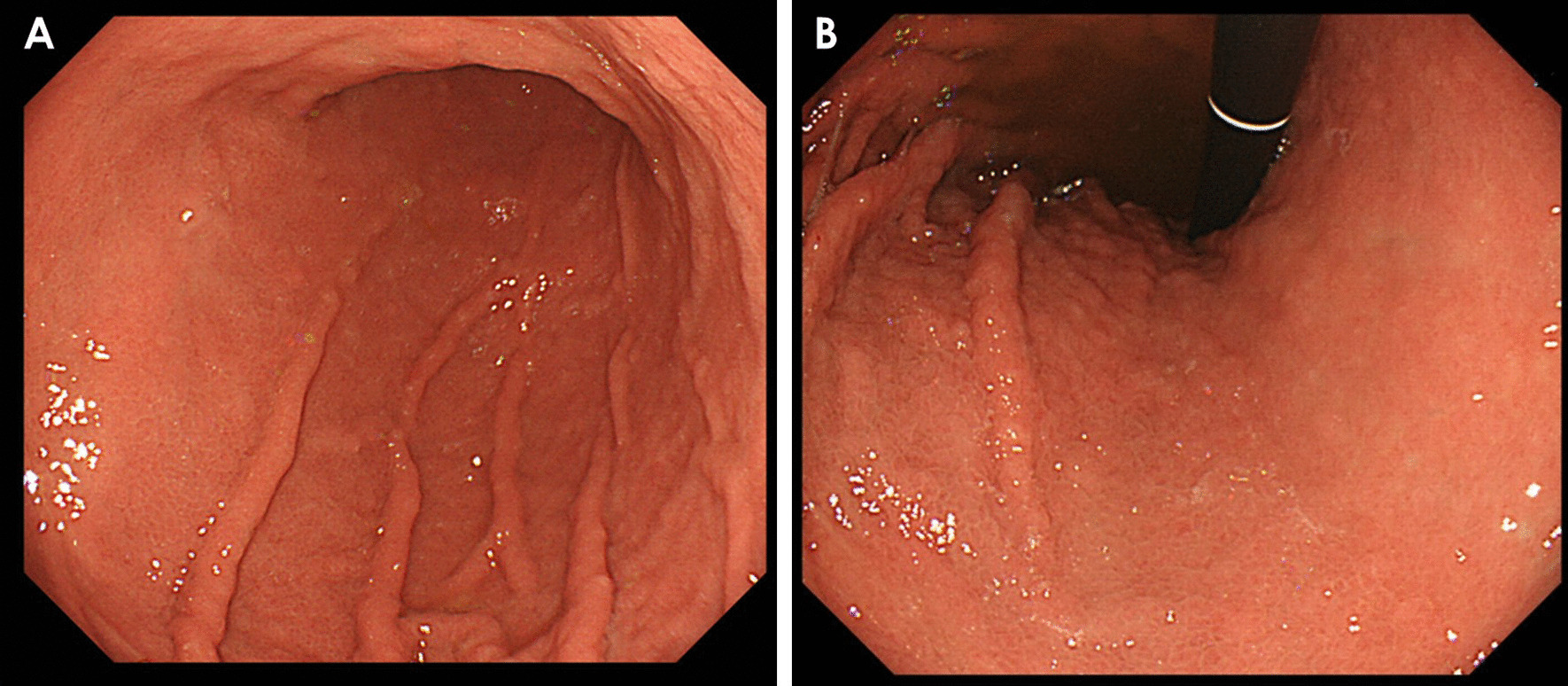
**A**, **B** The esophagogastroduodenoscopy showed atrophic gastritis with *Helicobacter pylori* infection. There were no forming giant gastric folds

**Fig. 3:**
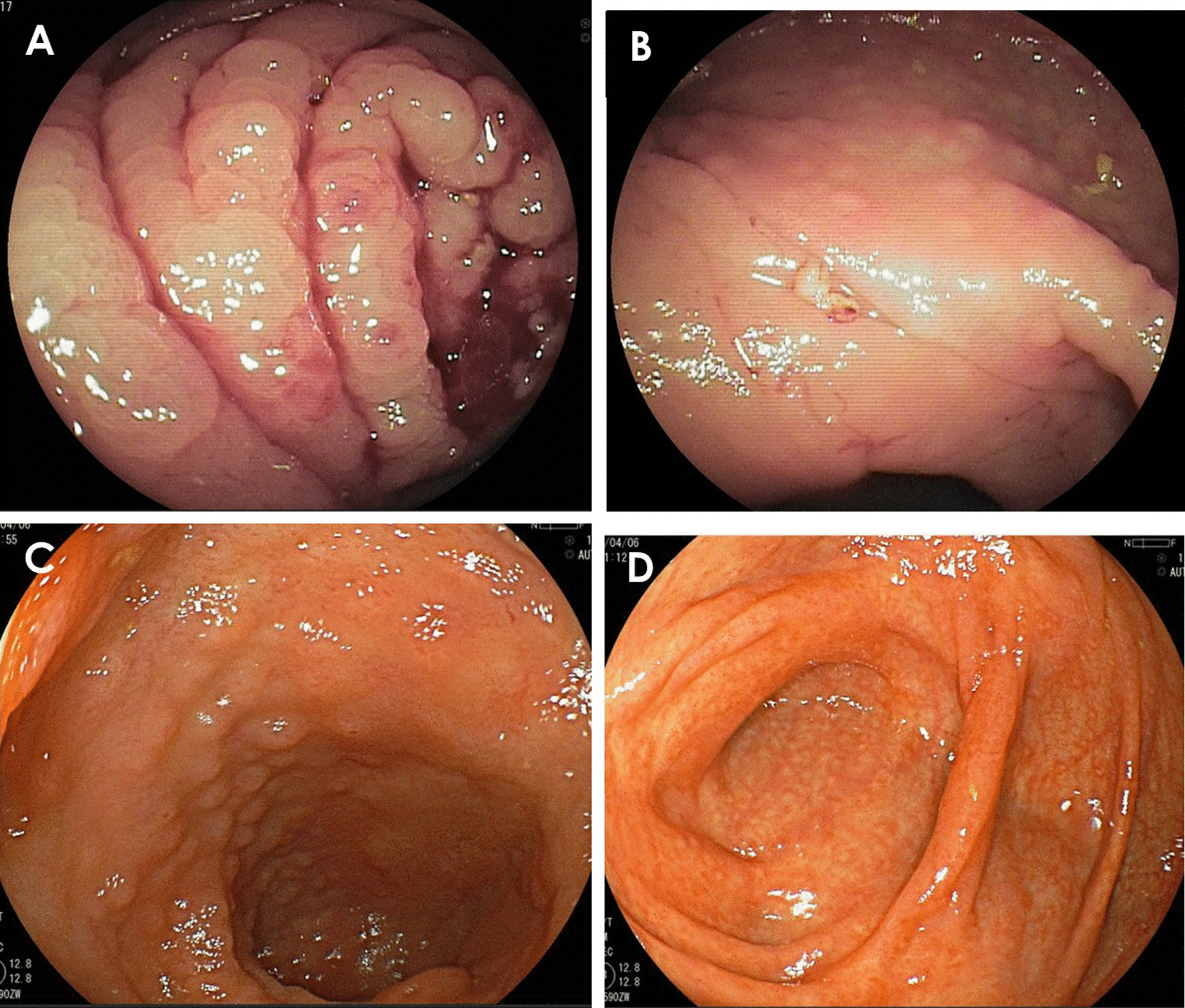
**A**, **B** Colonoscopy showed lymphatic hyperplasia in terminal ileum and cecum. **C**, **D** Colonoscopy showed improvement of lymphatic hyperplasia in the ileum and cecum

**Fig. 4:**
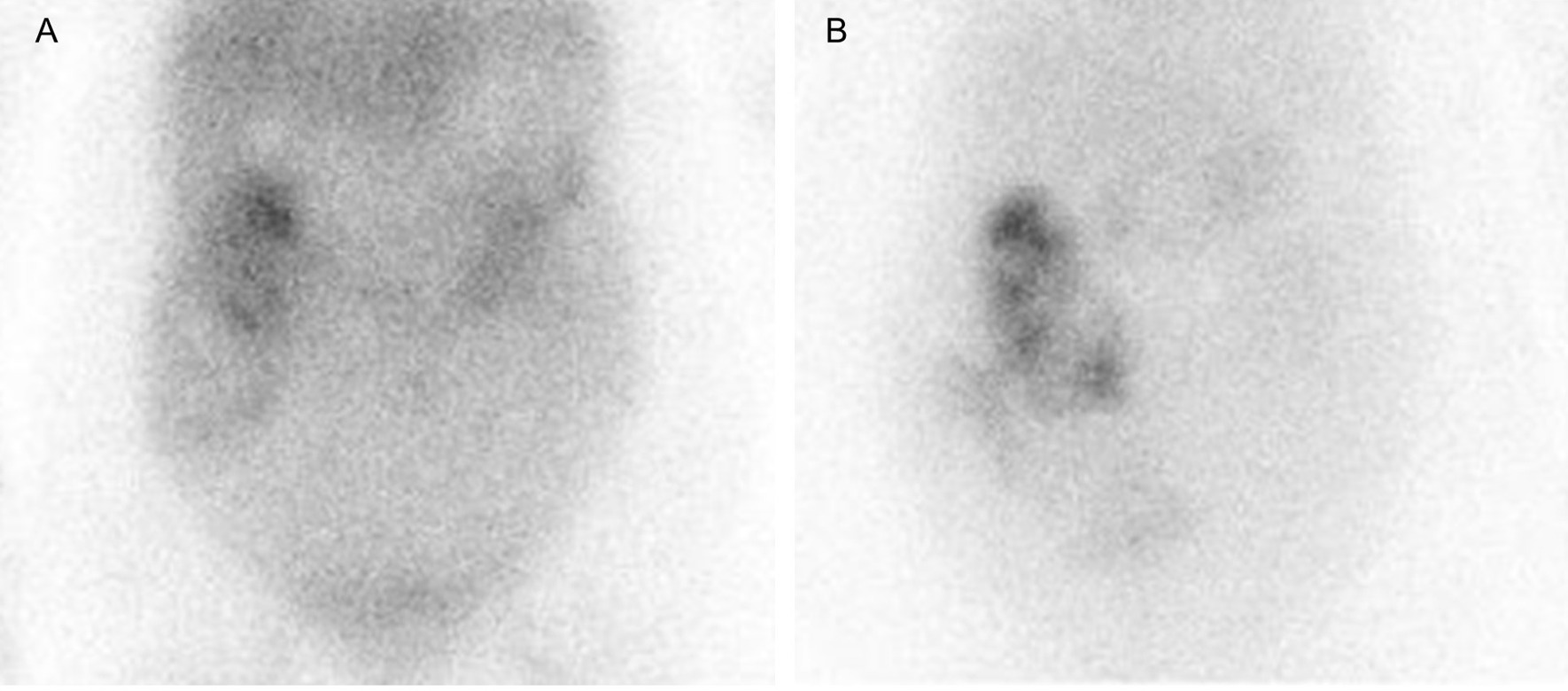
^99m^Tc-DTPA-binding human albumin scintigraphy. Protein leakage was shown from the cecum to the transverse colon

**Fig. 5:**
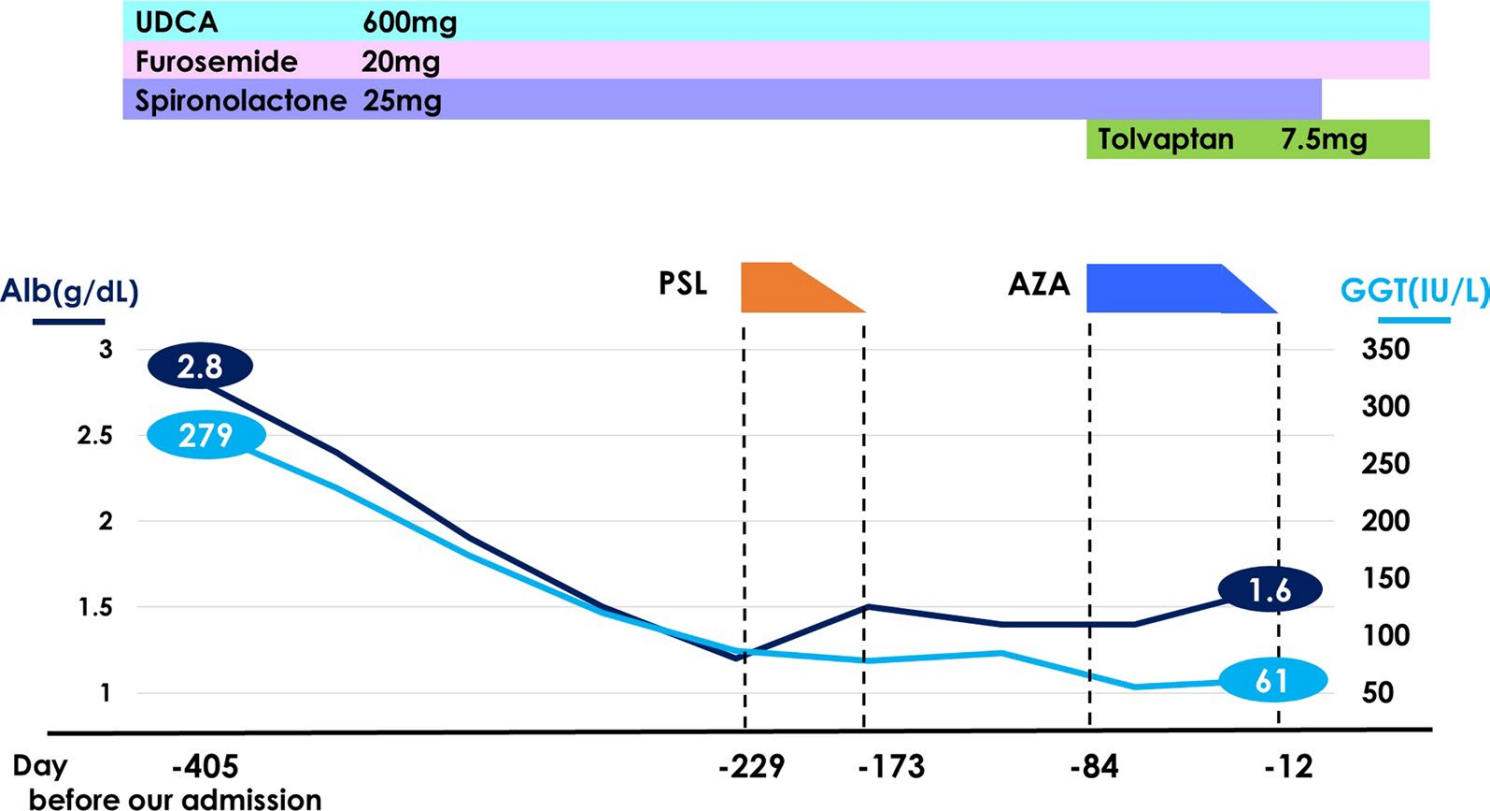
The time course of treatment at the previous hospital

A 71-year-old woman visited another hospital for an investigation of hypoalbuminemia, liver dysfunction, and systemic edema. The patient’s medical history included type 2 diabetes mellitus and hypertension, for which she had been undergoing treatment since the age of approximately 50 years. There was no remarkable family history, and the patient was a social drinker who never smoked cigarettes. The results of laboratory tests were as follows: total bilirubin (T-Bil), 0.4 mg/dL; aspartate aminotransferase (AST), 67 IU/L; alanine aminotransferase (ALT), 76 IU/L; alkaline phosphatase (ALP), 590 IU/L; gamma-glutamyl transpeptidase (GGT), 279 IU/L; and albumin (Alb), 2.8 g/dL. Anti-nuclear antibody was 1:80, and antimitochondrial antibody was 12.7 U/mL. Computed tomography (CT) showed no remarkable finding except for subcutaneous edema and ascites (Fig. 1A). Esophagogastroduodenoscopy (EGD) showed atrophic gastritis, and a biopsy of the gastric mucosa showed *H. pylori* infection; however, it did not reveal giant gastric folds; a characteristic feature of Menetrier's disease (Fig. 2A, B). Colonoscopy showed lymphatic hyperplasia in terminal ileum and cecum (Fig. 3A, B), and histology of the cecum showed proliferation of lymph vessels. Capsule and balloon endoscopies showed multiple angiodysplasia and lymphatic hyperplasia in the ileum. The fecal alpha-antitrypsin clearance test result was 170 mL/day (0.4–19.0 mL/day), and ^99m^Tc-diethylenetriaminepentaacetic acid-binding human albumin scintigraphy showed protein leakage from the cecum to the transverse colon (Fig. 4). Finally, the patient was diagnosed with PLGE complicated with aPBC. The time course of treatment at the previous hospital is shown in Fig. 5. Ursodeoxycholic acid (UDCA) was administered for aPBC. After administering UDCA, liver function rapidly improved; however, hypoalbuminemia and edema remained; hence, prednisolone 50 mg was started for PLGE. No improvement of symptoms was observed after prednisolone administration. After several weeks, azathioprine (AZA) 100 mg/day was started for PLGE, but it was not effective and was tapered and discontinued (Fig. 5). As the patient's edema did not improve after 6 months of hospitalization, she was transferred to our hospital for rehabilitation.

**Fig. 6:**
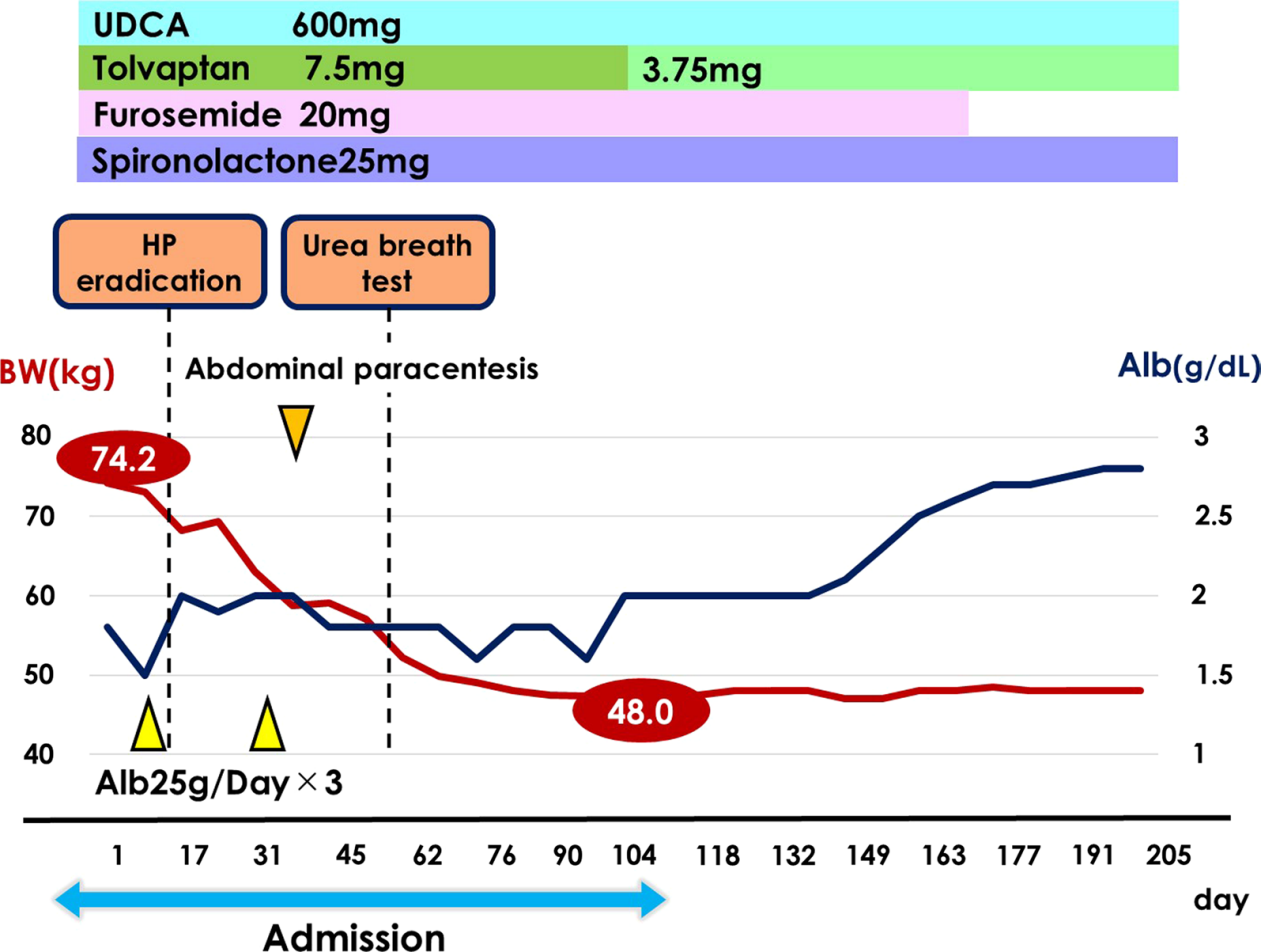
The time course of treatment at our hospital

At the time of admission, the patient’s height was 152 cm, body weight was 74.2 kg, and body mass index was 32.1 kg/m^2^. Physical examination revealed conjunctival anemia, a Levine II/VI systolic murmur, and anasarca. The patient was unable to walk independently due to severe leg edema. The results of the laboratory investigation on admission were as follows: C-reactive protein, 1.38 mg/dL; total protein, 5.7 g/dL; Alb, 1.8 g/dL; T-Bil, 0.2 mg/dL; AST, 8 IU/L; ALT, 3 IU/L; ALP, 411 IU/L; and GGT, 57 IU/L (Table 1). We continued the previous doctor’s prescription after the patient was transferred to our hospital, and albumin transfusion was performed several times. As hypoalbuminemia persisted after administering UDCA for aPBC, we considered that the occurrence of PLGE was not due to aPBC. The EGD performed at the previous hospital showed no suspicious findings for Menetrier's disease, and ^99m^Tc-labelled albumin scintigraphy showed no protein leakage from the stomach. We hypothesized that PLGE may have developed due to changes in the intestinal immune system associated with *H. pylori* infection, and we decided to eradicate *H. pylori*. Therefore, we performed *H. pylori* eradication by prescribing 1500 mg of amoxicillin, 400 mg of clarithromycin, and 40 mg of vonoprazan for 7 days. On day 45 after *H. pylori* eradication, we performed a urea breath test and confirmed that *H. pylori* was successfully eradicated. The time course of treatment at our hospital is shown in Fig. 6. Except for *H. pylori* eradication therapy, the patient's treatment was unchanged. Diuretics were continued at the same dose as in the previous hospital. The hypoalbuminemia improved slowly, but the edema improved markedly. The patient continued physical therapy and was able to walk independently. Therefore, the patient was discharged on day 107 after hospitalization at our hospital. Her body weight had decreased from 74.2 kg at presentation to 48.0 kg at discharge (26.2 kg loss). After discharge, abdominal CT revealed complete disappearance of ascites and subcutaneous edema (Fig. 1B). Follow-up colonoscopy showed improvement of ileal and cecum lymphatic hyperplasia (Fig. 2C, D). Albumin recovered to 3.4 g/dL, 1 year after *H. pylori* eradication. After approximately 4 years of follow-up, ascites and edema have not recurred after discontinuing diuretics, and Alb was sustained over 3.0 g/dL with no adverse events.

**Table 1:** Laboratory examination on admission

*CBC*		*Biochemistry*				
WBC	39 × 10^2^/μL	TP	5.7 g/dL	T.Cho	322 mg/dL	
Neut	64%	Alb	1.8 g/dL	TG	225 mg/dL	
Mono	3%	T.Bil	0.2 mg/dL	LDL-cho	242 mg/dL	
Eosin	0%	AST	8 IU/L	HDL-cho	23 mg/dL	
Baso	1%	ALT	3 IU/L	CRP	1.38 mg/dL	
Lym	34%	ALP	411 IU/L	HBsAg	Negative	
RBC	245 × 10^4^ /μL	γ-GTP	57 IU/L	HCVAb	Negative	
Hb	8.3 g/dL	LDH	127 IU/L	IgA	544.7 mg/dL	
Plt	26.4 × 10^4^ /μL	AMY	40 IU/L	IgM	171.2 mg/dL	
*Coagulation test*		CK	27 IU/L	IgG	903.6 mg/dL	
PT ratio	1.04	BUN	14.9 mg/dL	BTR	7.52	
PT activity value	91 %	CRE	0.4 mg/dL	TSH	0.97 μIU/mL	
APTT	29 s	Na	140 mEq/L	FT3	2.1 ng/dL	
FDP	14 μg/mL	K	3.2 mEq/L	FT4	1.08 pg/mL	
D-dimer	5.4 μg/mL	Cl	103 mEq/L	ANA	40 fold	
		Glu	177 mg/dL	AMA2	12.9 IU/mL	
		ChE	290 IU/L	BNP	129.7 pg/mL

## Discussion and conclusions

In the present case, following *H. pylori* eradication, clinical symptoms, such as edema and ascites, extremely improved and hypoalbuminemia gradually recovered, thereby confirming that PLGE can be improved by *H. pylori* eradication. PLGE is a syndrome with a chief complaint of hypoalbuminemia, which occurs due to protein leakage in the gastrointestinal tract. The course of PLGE varies to a great extent, which includes systemic diseases, such as autoimmune disease, heart disorder, as well as gastrointestinal disease. There are three major mechanisms of protein leakage in PLGE. First, in the case of increased capillary endothelial permeability, such as autoimmune diseases or eosinophilic gastroenteropathy, the amount of protein in the interstitial space excessed the drainage ability of the lymphatic system. Second, in patients with increased lymphatic pressure, the lymphatic vessel ruptures into the intestinal lumen, allowing decompression and retrograde drainage of systemic lymph. Third, breakdown of the mucosal barrier, like mucosal erosion or ulcers, allows easy passage of interstitial protein into the intestine, for example, in conditions, such as inflammatory bowel disease (IBD), infection, or neoplasia. To accurately determine the cause of PLGE, comprehensive examinations are required, such as contrast-enhanced systemic CT, serum autoantibodies, upper and lower intestinal endoscopy, echocardiography, and so on. A diagnosis of PLGE can be made by 24-h fecal alpha-1 antitrypsin clearance [1]; however, this cannot identify the site of albumin leakage. In contrast, ^99m^Tc-labelled albumin scintigraphy is useful for detecting the location of albumin leakage [2–5], which may provide insights on the site of biopsy for histopathological investigation.

Considering autoimmune diseases, rheumatoid arthritis and SLE are relatively common causes of PLGE, and systemic sclerosis is also recognized as a cause of PLGE [6, 7]. The prevalence of PLGE with SLE has been reported to range from 0.94 to 7.5% [2, 3, 8]. There is no previous report of PLGE complicated with aPBC. The location of leakage can generally range from the stomach to the colon. In the present case, PLGE was complicated with aPBC, and the location of leakage was the terminal ilium and intestine. The characteristic histopathological findings of PLGE are inflammatory cell infiltration, edematous interstitial tissue, atrophied villi, and lymphangiectasis. Regarding the pathology of PLGE associated with SLE, it has been reported that inflammatory cell infiltration was observed in 73% of patients, lymphangiectasis in 15% of patients, vasculitis in 2% of patients, and normal cellular morphology in 20% of patients [2].

The first-line treatment of PLGE is prednisolone. However, the efficacy of monotherapy with prednisolone is sometimes insufficient to suppress the disease. Therefore, various additional immunosuppressive agents are used to achieve a successful response. AZA, cyclosporin A, cyclophosphamide, and methotrexate are prescribed as additional immunosuppressive agents. Regarding PLGE in SLE, more than 60% of patients eventually require additional immunosuppressants, as part of combination therapy with prednisolone, to maintain clinical remission.

Menetrier’s disease is known as hypoproteinemic hypertrophic gastropathy, characterized by giant rugal folds and excessive mucous production with protein loss. Menetrier's disease has been previously reported to be associated with *H. pylori* infection [9]. The endoscopic findings of PLGE with *H. pylori* infection are mainly divided into Menetrier’s disease, diffuse varioliform gastritis, or rarely cap polyposis. Previous report studies showed that *H. pylori* eradication completely resolved PLGE in Menetrier’s disease, hypertrophic lymphocytic gastritis, or erosive gastritis [10, 11]. The mechanism by which *H. pylori* infection causes PLGE is not fully understood; however, a defect in the epithelial tight junction has been suspected previously. A recent report showed PLGE due to *H. pylori* infection without giant rugal folds, erosion, or polyposis [12], suggesting a defect in the epithelial tight junction caused by *H. pylori* infection in the intestine. In our present case, EGD showed no typical findings of PLGE in the stomach, and the terminal ileum showed lymphatic hyperplasia, which may have been influenced by *H. pylori* to intestinal tight junction protein.

Recently, the role of regulatory T cells (Tregs) in maintaining homeostasis in intestinal immunoregulatory mechanisms has received increasing attention. Tregs are expected to be applied for treating IBD by suppressing excessive immune responses in the intestine [13]. Tregs are also activated during *H. pylori* infection, which is involved in immune tolerance [14]; however, a mechanism of persistent infection in which *H. pylori* is not eliminated has been reported. However, there are no reports concerning PLGE and Tregs. Therefore, it is necessary to accumulate further clinical evidence regarding PLGE to clarify the details of this disease. In the present case, activation of Treg may have caused abnormalities not only in the stomach but also in the intestinal immune control mechanism, which may have contributed to the development of PLGE, and *H. pylori* eradication improved PLGE. We consider that *H. pylori* eradication also improved the inflammation of the gastrointestinal tract, resulting in improved lymphatic hyperplasia of the cecum.

In conclusion, this is the first report wherein eradication of *H. pylori* improved PLGE complicated with aPBC, refractory to prednisolone and other immunosuppressants. Our results suggest that a clinical trial of *H. pylori* eradication is warranted in patients with PLGE accompanied by *H. pylori* infection.

## Data Availability

Data sharing is not applicable to this article as no datasets were generated or analyzed in this study.

## Abbreviations

Alb: Albumin
ALP: Alkaline phosphatase
ALT: Alanine aminotransferase
aPBC: Asymptomatic primary biliary cholangitis
AST: Aspartate aminotransferase
AZA: Azathioprine
CT: Computed tomography
EGD: Esophagogastroduodenoscopy
GGT: Gamma-glutamyl transpeptidase
*H*. *pylori*: *Helicobacter pylori*
IBD: Inflammatory bowel disease
PLGE: Protein-losing gastroenteropathy
SLE: Systemic lupus erythematosus
T-Bil: Total bilirubin
Tregs: Regulatory T cells
UDCA: Ursodeoxycholic acid
